# A Systematic Review of Investigations into Functional Brain Connectivity Following Spinal Cord Injury

**DOI:** 10.3389/fnhum.2017.00517

**Published:** 2017-10-25

**Authors:** Alkinoos Athanasiou, Manousos A. Klados, Niki Pandria, Nicolas Foroglou, Kyriaki R. Kavazidi, Konstantinos Polyzoidis, Panagiotis D. Bamidis

**Affiliations:** ^1^Laboratory of Medical Physics, Faculty of Medicine, School of Health Sciences, Aristotle University of Thessaloniki, Thessaloniki, Greece; ^2^First Department of Neurosurgery, AHEPA University Hospital, Aristotle University of Thessaloniki, Thessaloniki, Greece; ^3^Department of Biomedical Engineering, School of Life and Health Sciences, Aston University, Birmingham, United Kingdom

**Keywords:** brain connectivity, brain network, cortical connectivity, cortical network, maladaptive plasticity, network reorganization, sensorimotor network, spinal cord injury

## Abstract

**Background**: Complete or incomplete spinal cord injury (SCI) results in varying degree of motor, sensory and autonomic impairment. Long-lasting, often irreversible disability results from disconnection of efferent and afferent pathways. How does this disconnection affect brain function is not so clear. Changes in brain organization and structure have been associated with SCI and have been extensively studied and reviewed. Yet, our knowledge regarding brain connectivity changes following SCI is overall lacking.

**Methods**: In this study we conduct a systematic review of articles regarding investigations of functional brain networks following SCI, searching on PubMed, Scopus and ScienceDirect according to PRISMA-P 2015 statement standards.

**Results**: Changes in brain connectivity have been shown even during the early stages of the chronic condition and correlate with the degree of neurological impairment. Connectivity changes appear as dynamic post-injury procedures. Sensorimotor networks of patients and healthy individuals share similar patterns but new functional interactions have been identified as unique to SCI networks.

**Conclusions**: Large-scale, multi-modal, longitudinal studies on SCI patients are needed to understand how brain network reorganization is established and progresses through the course of the condition. The expected insight holds clinical relevance in preventing maladaptive plasticity after SCI through individualized neurorehabilitation, as well as the design of connectivity-based brain-computer interfaces and assistive technologies for SCI patients.

## Introduction

### Rationale

Complete or incomplete spinal cord injury (SCI) results in varying degree of motor, sensory and autonomic impairment and long-lasting, often irreversible disability (Anderson et al., 2011). This impairment results from injury to efferent and afferent neural pathways but even in complete SCI, a true dissection of the spinal cord is uncommon (Nardone et al., 2015). The anterior and lateral corticospinal tracts, the lateral and ventral spinothalamic tracts, the ventral and dorsal spinocerebellar tracts, as well as the sympathetic and parasympathetic fibers located within the spinal cord are the major pathways responsible for reciprocal communication of central and peripheral nervous systems. How does disconnection of this reciprocal communication affect brain function and connectivity has not yet become so clear. While anatomical white matter connections of cortical sensorimotor areas and subcortical nodes or connections between homologous sensorimotor cortical areas, that are effected through the corpus callosum directly (Zarei et al., 2006) or indirectly (Meyer et al., 1998), are not primarily damaged, changes in brain organization and structure have indeed been associated with SCI and have been extensively studied and reviewed (Freund et al., 2013; Nardone et al., 2013).

Some of these reported alterations can be considered structural changes, including atrophy of the afferent nerve pathways, microstructural changes of efferent axons and regions of the sensorimotor cortex (Freund et al., 2013; Nardone et al., 2013). These changes have been observed to occur even in the first months after the injury and the rate that they evolve is associated with poorer prognosis for neurological rehabilitation; to the point that they can be considered as neuroimaging biomarkers (Freund et al., 2013). Plastic changes of neuronal circuits occur after SCI at multiple levels, mainly in the cortical representation of motor and sensory areas and are related to the severity of the injury. The exact pathophysiological mechanism is not known, but such aberrant reorganization may have pathological consequences (Nardone et al., 2013). This maladaptive plasticity occurring after SCI constitutes an issue that has begun to raise many questions for researchers as to its origin and the possibility of suspension (Tidoni et al., 2015). Moreover, among biomarkers, significant relationship has been revealed between cortical reorganization and neuropathic pain, which is now considered almost a definite indicator of maladaptive plasticity (Pascoal-Faria et al., 2015). Such biomarkers of aberration already fall under the targeted focus of interventions to early prevent maladaptive plasticity (Zantedeschi and Pazzaglia, 2016). Ultimately, what presents as an important issue for investigation is the possibility of neurological rehabilitation of SCI patients and whether these studies dealing with the functional reorganization of the brain circuits and spinal cord, can provide both useful knowledge and practical solutions (Alam et al., 2016).

### Objectives

The scientific community’s knowledge regarding brain connectivity changes following SCI is overall lacking in comparison. Relevant studies have been appearing during the last decade and little only effort has been made into synthesizing this body of literature. The goal of the present study is to conduct a systematic review of articles regarding investigations of functional brain networks after SCI and present a synthesis of the results in a coherent fashion.

### Research Question

Such a review has not yet been conducted and is expected to provide insight into the pathophysiological processes that occur in the brain after the disruption of the sensorimotor pathways. Existing literature does not yet portray how reorganization of functional brain networks is installed and progresses through the course of the condition. Specific research questions include how and why reorganization at the level of functional brain connectivity is established and evolves and whether it is adaptive or maladaptive. The review was conducted by searching into major databases of peer-reviewed literature and synthesis of retrieved journal articles.

## Material and Methods

### Study Design and Search Strategy

The study was performed according to PRISMA-P 2015 statement standards (Moher et al., 2015). The online databases PubMed, Scopus and ScienceDirect were systematically searched for relevant studies in June 2016; the search was updated in July 2017. The search was performed using the terms “brain connectivity”, “brain network”, “cortical connectivity” and “cortical network” combined with the term “SCI”. No publication year criteria were set. Duplicate entries were then removed and two independent researchers screened and selected articles for relevancy based on title, abstract and full text. Furthermore, relevant articles were hand-searched and screened through the references of retrieved original and review studies. The percentage of inter-rate agreement of the selection by the two researchers was calculated. In disagreement regarding specific selections, a third independent researcher reviewed the case and finally decided for or against relevancy of the unique entry.

### Data Sources, Studies Sections and Data Extraction

For the synthesis, our inclusion and exclusion criteria were set as follows: only peer-reviewed original studies providing calculative methodology to estimate brain connectivity on human SCI patients were included; no threshold was set for number of patients but uncontrolled case reports were excluded. Furthermore, theoretical mention to brain networks or discussion about networks was not enough for a article to be included in the review. Brain activation studies with no estimation of connectivity of activated areas, as well as intrinsic spinal cord connectivity studies were also excluded and so were studies on animal models. Non-peer reviewed articles (e.g., in magazines) were excluded. Conference articles were also excluded if the results were presented in a subsequent journal article by the same research team, to avoid over-presentation bias. Review articles were also excluded. If full text of an article was unavailable, the authors were contacted by email.

## Results

### Systematic Search Results

**Figure 1:**
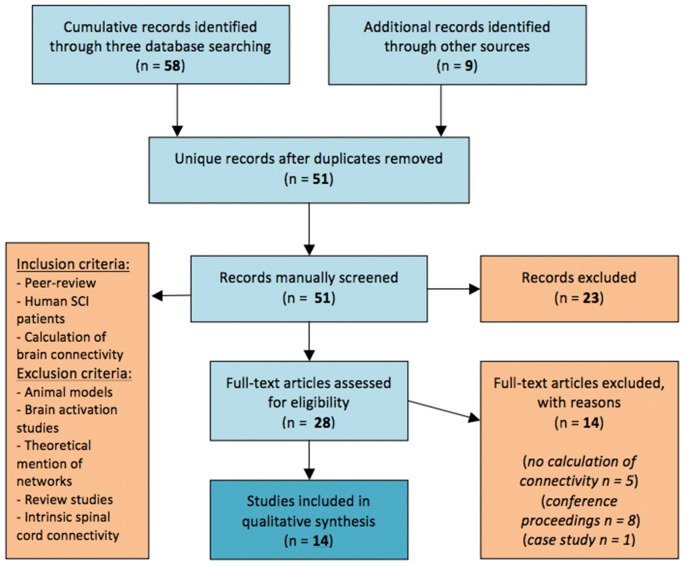
Flow diagram of the method followed through the systematic review according to PRISMA standards.

The initial search in all three databases and through other sources yielded 65 titles, while two more titles were recovered during the update. After removal of duplicate entries from this list, we derived 51 unique entries that were all manually screened for relevance. The percentage of inter-rater agreement was 96.08% with a Cohen’s kappa (McHugh, 2012) of *k* = 0.920 and a 95% Confidence Interval (CI) of 0.82–1.03. A total of 28 articles were selected as relevant at this step. According to our inclusion and exclusion criteria (Figure 1), those 28 articles were assessed for eligibility; five of them were excluded for lack of providing reproducible calculative methodology to estimate brain connectivity and eight articles were excluded as being conference articles because of a subsequent journal publication by the same team also covered their results. A single case study was also excluded from the presentation of the synthesis results.

As a result, a total of 14 articles were included in the synthesis (see Figure 1); seven articles reported functional Magnetic Resonance Imaging studies (fMRI), while seven articles reported Electroencephalography (EEG) studies that all came from the same research group (Table 1).

**Table 1:** Published journal articles on brain connectivity after spinal cord injury (SCI) in human patients that were included in the qualitative synthesis.

References	Modality	No. of patients	No. of controls	Time post-injury (months)	Connectivity analysis
Astolfi et al. (2007)	EEG	5	6	N/A	ROI/MI
De Vico Fallani et al. (2007a)	EEG	5	5	N/A**	ROI/MI/GA
De Vico Fallani et al. (2007b)	EEG	5	5	N/A**	ROI/MI/GA
Mattia et al. (2009)	EEG	5	5	19.4 ± 7.2	ROI/MI/GA
Sinatra et al. (2009)	EEG	5	5	N/A**	ROI/MI/GA
Astolfi et al. (2010)	EEG	5	5	18.4 ± 6	ROI/MI/TV
De Vico Fallani et al. (2011)	EEG	5	5	N/A**	ROI/MI/GA
Hou et al. (2014a)	fMRI	25	25	9.3 ± 2.9 weeks*	SB/RS
Min et al. (2015a)	fMRI	18	18	>3	SB/RS
Min et al. (2015b)	fMRI	20	20	>3	ROI/RS/GA
Hou et al. (2016)	fMRI	25	25	9.2 ± 3.5 and 8.8 ± 2.6 weeks*	SB/RS
Oni-Orisan et al. (2016)	fMRI	11	9	>24	SB/RS
Kaushal et al. (2017a)	fMRI	15	15	>24***	ROI/RS/GA
Kaushal et al. (2017b)	fMRI	15	15	>24***	ROI/RS/GA

EEG, electroencephalography; fMRI, functional Magnetic Resonance Imaging; GA, graph analysis; MI, motor imagery; SB, seed-based; ROI, Regions of Interest-based; RS, resting-state; TV, Time-varying; *same subjects, grouped by recovery, **,***same patient group.

### Functional Magnetic Resonance Imaging Studies

The degree of neurological impairment has been shown to correlate with alterations of brain function and connectivity that appear even during early phases of SCI as Hou et al. (2014a) have demonstrated in a resting-state (RS) fMRI study on 25 SCI patients 2 months after the injury, at a sub-acute phase. The authors report that SCI patients present network alterations negatively correlating with the motor sub-score of the International Standards for Neurological Classification of SCI. In addition, they presented decreased amplitude of low frequency fluctuations (ALFF) in primary sensorimotor areas (M1) bilaterally and increased ALFF in the cerebellum (CB) bilaterally and right orbitofrontal cortex (OFC). The connectivity analysis was performed on a seed-based (SB) level, whereas the five regions that were chosen as seeds where those identified in ALFF analysis. The main findings of the connectivity analysis included: (1) decreased inter-hemispheric Functional Connectivity (FC) between bilateral M1 areas; and (2) increased FC within the areas of the motor network of each individual hemisphere, namely M1, premotor cortex (PMC), supplementary motor area (SMA), thalamus (TH) and CB. Moreover, the authors calculated the average FC in areas with abnormal alterations and checked for correlation with disease duration and severity of patient symptomatology. They found that the intra-hemispheric increase between M1, SMA and CB was the element that also significantly correlated with higher degree of impairment (but not with the disease duration within the limits previously mentioned; Table 2).

**Table 2:** Main neurophysiological findings reported by fMRI studies of functional brain connectivity after SCI.

References	Main reported findings	Injury phase	Injury outcome
Hou et al. (2014a)	- ↓ inter-hemispheric FC between bilateral M1 areas - ↑ intra-hemisperic FC between M1, PMC, SMA, TH and CB o between M1, SMA and CB: correlated(+) with impairment	subacute	incomplete
Hou et al. (2016)	- poor recovery: ↓ FC between o bilateral SMAs o right M1 and right SMA and PMC	subacute	incomplete
	- good recovery: ↑ FC between o bilateral SMAs o right M1 and right SMA and PMC o left M1 and right SMA		
Min et al. (2015a)	- ↑ FC between S1, SMA and BG - FC between S1 and S2	subacute	incomplete
Min et al. (2015b)	- characteristic path length consistently ↑ in SCI - no other significant differences in graph analysis between SCI and Healthy	subacute	incomplete
Oni-Orisan et al. (2016)	- overall ↓ FC - ↓ FC between M1 and S1 and other nodes (intra- and inter-hemispheric) - ↑ FC between left S1 and bilateral TH	chronic	complete
Kaushal et al. (2017a)	- overall ↓ FC whole brain subnetwork - FC midline sensorimotor network and left CB	chronic	complete
Kaushal et al. (2017b)	- ↑ network modularity - local efficiency - no difference in global efficiency	chronic	complete

These alterations also seem to hold some importance in prognosis for SCI patients, as the same group of authors showed in another study (Hou et al., 2016). Using the same experimental paradigm, but differentiating between patients that showed good recovery vs. patients that showed poor recovery at 6 months after the injury, they retrospectively searched for predictors of the recovery degree. Patients with poor recovery at 6 months (Hou et al., 2016) seemed to show decreased RS FC between bilateral SMAs and between right M1 and right SMA and PMC at the original fMRI measurements performed at the 2 months post injury (Hou et al., 2014a) compared to healthy controls. On the other hand, patients with good recovery at 6 months (*n* = 10) showed increased FC between all those areas and between right SMA and left M1 when compared to healthy controls. They also showed increased FC in those areas when compared to patients with poor recovery (*n* = 15). Their findings suggest that adaptive increase of FC strength within the motor network at the sub-acute phase of SCI could be a predictor of long-term neurological prognosis for SCI patients.

Another RS fMRI study that attempted to investigate changes of brain FC after a SCI evaluated 18 patients with incomplete injury at the cervical section of the spinal cord (cervical injury), at least 3 months after the injury (Min et al., 2015a). The authors demonstrated increased RS FC in SCI patients between primary somatosensory area (S1), SMA and basal ganglia (BG). On the other hand, the authors demonstrated decreased RS FC between S1 and secondary somatosensory area (S2) for SCI patients compared to controls. The authors summarize their findings by denoting that motor components of the functional networks were found to present increased FC while sensory components presented decreased FC when compared to healthy controls. This finding is attributed to a network attempt to compensate for motor deficits (an adaptive effect of efferent components) and also a lack of any such mechanism for sensory deficits (a maladaptive effect of afferent components). The same group of authors also performed a graph analysis (GA) on the connectivity patterns that they measured on the same group of patients (Min et al., 2015b). They calculated graph properties of the RS networks over 45 Regions of Interest (ROIs) in each hemisphere, including clustering coefficient, characteristic path length, global efficiency and small-world-ness. No significant changes were identified except for the characteristic path length that was measured consistently higher in SCI patients when compared to healthy controls. The authors suggest that hub analysis of such findings could possibly provide answers regarding to neuronal plasticity following SCI.

Another study suggests that the continued disruption of sensorimotor pathways during chronic phases of complete SCI causes a de-organization of the RS functional networks, as this is measured by an overall decrease in FC (Oni-Orisan et al., 2016). The authors also obtained RS fMRI from 11 SCI patients with complete cervical injury after at least 2 years post-injury. They defined bilateral M1 and S1 as seed areas and measured significant alterations of the sensorimotor network. M1 and S1 showed decreased RS FC between them and other adjacent cortical sensorimotor nodes both in the same hemisphere and inter-hemispherically. The authors also demonstrated that connectivity with deeper structures does not remain unaltered as well, as shown by an increase of FC between left S1 and bilateral TH. They suggest that the results of their study are the effects of inherent neural plasticity and dynamic reorganization after an SCI. Since the patients involved all suffered from complete cervical injury these results are observed in the absence or a residual reciprocal sensorimotor communication. The authors also analyzed their recordings on 15 patients with complete cervical SCI with GA of the FC using network-based statistics over 58 ROIs in each hemisphere (Kaushal et al., 2017a). They observed diminished RS connectivity strengths of a whole-brain sub-network in SCI patients compared to healthy controls. They moreover observed enhanced connectivity strengths in a sub-network containing midline sensorimotor cortex and left CB of SCI patients, attributing it to a higher degree of neuronal intercommunication of those areas due to the injury. Kaushal et al. (2017b) also applied large-scale GA to their data to further analyze the RS networks of the SCI patients in comparison to those of healthy individuals. They calculated correlation between pairs the already defined ROIs and then calculated modularity, as well as local and global efficiency. The authors observed increased modularity of the RS networks of SCI patients (nine modules compared to seven of those in healthy ones) and decreased local efficiency (a statistical significant observation) of the nodes in SCI patients but not significant differences in global efficiency of the network.

### Electroencephalography Studies

EEG has been used as a neurophysiological modality to investigate brain networks of SCI patients in a non-RS, employing a motor imagery (MI) task (Table 3). In this series of publications, the patients were asked to repetitively imagine moving (to attempt to move) their disabled right foot while protruding their lips (which they were able to move), under high-resolution EEG recording. The EEG recording was then projected onto a cortical model of the patients’ brain, which was attained by MRI scan and then parcelated by six sensorimotor related ROIs (on each hemisphere) and the frequency bands that were analyzed were theta (4–7 hz), alpha (8–12 hz), beta (12–30 hz) and gamma (30–40 hz; Astolfi et al., 2007, 2010; De Vico Fallani et al., 2007a,b, 2011; Mattia et al., 2009; Sinatra et al., 2009).

**Table 3:** Main neurophysiological findings reported by electroencephalographic studies of functional cortical connectivity after SCI (chronic complete injury at the cervical spine level).

References	Main reported findings	Healthy	SCI chronic complete
Astolfi et al. (2007)	- CMA as an important information hub	√	√
	- CMA and right foot M1 → bilateral SMAs	√	
	- SMAs receive wider inflow		√
De Vico Fallani et al. (2007a)	- ↑ network fault tolerance during motor imagery of paralyzed foot		√
	- ↑ of local efficiency		√
De Vico Fallani et al. (2007b)	- CMA important hub of beta rhythm networks	√	√
	- foot M1 notable outflow during motor imagery	√	
	- SMA notable outflow during motor imagery		√
Mattia et al. (2009)	- similar patterns of connectivity for healthy and SCI	√	√
	- new unique interactions in SCI		√
	o CMA and SMA → ipsilateral SPC		
	o Bilateral foot M1 ←→ SMA		
Sinatra et al. (2009)	- network separated into two dense clusters		
	o CMAs—SMAs		√
	o PMCs—right foot M1		
Astolfi et al. (2010)	- ↑ temporally dynamic cortical networks		√
	- ↑ involvement of the parietal cortex around motor		√
	- imagery onset		
De Vico Fallani et al. (2011)	- no differece in redundancy (theta band)	√	√
	- ↑ communication between closest cortical areas		√

In the study by Astolfi et al. (2007), five SCI patients with complete cervical injury participated in the experiment. Brain connectivity was calculated using Partial Directed Coherence. The study concluded that in general, MI driven cortical networks supporting the will to move of SCI patients are larger than of healthy participants (who performed motor execution), while no specific frequency band presents greater causality for the task investigated. Cingulate motor area (CMA) was identified as an important information hub as, along with foot M1 area, was found able to outflow information to SMA bilaterally in healthy individuals—while in SCI patients the SMAs received information from a wider array of nodes.

The brain network properties of chronic complete SCI patients have also been investigated with GA. In the study by De Vico Fallani et al. (2007a) Directed Transfer Function was used to calculate cortical connectivity over 12 ROIs on five SCI patients with the same experimental procedure. The authors found that SCI patients MI networks present higher fault tolerance during attempted movement of the paralyzed foot, as an internal organization characteristic, represented by an increase of the networks’ local efficiency. The authors re-affirm the role of CMA an important information hub in the beta frequency band (De Vico Fallani et al., 2007b) in both healthy and SCI networks, while SMA show a notable outflow in SCI patients and M1 foot area in healthy individuals. Further studying the topological properties of these networks by identifying community structures within them (by Markov Clustering method) revealed large differences between healthy and SCI patient networks (Sinatra et al., 2009). SCI networks in the alpha frequency seem functionally separated in two dense clusters (CMAs/SMAs and PMCs/right foot M1), something that is suggested by the authors to strengthen the original idea of a compensatory mechanism regarding fault tolerance in the network (Sinatra et al., 2009) as a result of the long neural tracks disconnection. Also supporting of this idea, is that in lower EEG spectra (theta band), no difference in redundancy index was shown between the networks of SCI patients and healthy individuals while a higher degree of communication between closest cortical areas in SCI patient networks was demonstrated (De Vico Fallani et al., 2011).

The MI networks of SCI patients and healthy individuals share certain similar patterns of connectivity but there are new functional interactions that were identified as unique to SCI patients, in another study on five SCI patients with complete injury, using the same experimental paradigm (Mattia et al., 2009). These unique interactions include an inflow of information from the ipsilateral CMA and SMA to the superior parietal cortex (SPC) and exchange of information between bilateral primary motor foot area and SMA (Mattia et al., 2009). A dynamic time-varying (TV) estimation of functional cortical connectivity using Partial Directed Coherence was also demonstrated (Astolfi et al., 2010). This study also elaborated on unique interactions of SCI networks, as it further showed larger temporally dynamic cortical networks for the SCI patients compared to healthy individuals and a greater involvement of the parietal cortex around MI onset (Astolfi et al., 2010).

## Discussion

Changes in brain connectivity have been shown to appear even during the early stages of the chronic condition and to correlate with the degree of neurological impairment (Hou et al., 2014a). In a non-connectivity based fMRI study on a mixed group of patients (with both complete and incomplete SCI patients) within the first month after the injury, altered spontaneous RS brain activations were recorded in almost all cortical and sub-cortical sensorimotor areas (Zhu et al., 2015). In the study conducted by Hou et al. (2014a), using fMRI on SCI patients 2 months post-injury, the above described network alterations also correspond to structural changes as well, namely important gray matter atrophy at the sensorimotor cortical areas and pathways (Hou et al., 2014b). It can be speculated that the same mechanisms that drive structural alterations after SCI are also responsible for network adaptivity as well. Therefore, FC changes can be theorized to be dynamic post-injury procedures (subject to evolution across time and the progression of the injury-associated neurological impairments). While this dynamic procedure has not been directly demonstrated in a human longitudinal study, relevant evidence can be derived from as study of this type on rhesus monkeys by Rao et al. (2016). In this animal model, RS fMRI of rhesus monkeys with planned incomplete SCI (hemi-transection) was studied and recordings were made at pre-injury and 4, 8 and 12 months post-injury time points. While the study showed initially an increased FC between multiple major sensorimotor nodes such as primary sensorimotor cortices, SMA and putamen, FC tended to gradually approach baseline levels in most of the aforementioned areas at 12 weeks post-injury. The initial increase could possibly correspond to the reported increased intra-hemispheric FC in humans in the study by Hou et al. (2014a). Moreover, the degree of recovery at mean 12 weeks post-injury in human patients was also associated with increased FC between SMA and PMC (Hou et al., 2016).

Although progression of reorganization has been somewhat portrayed, the same cannot be said with regards to the underlying mechanism of reorganization. Although no clear conclusions can be drawn yet, there are indications that the disruption of reciprocal pathways can cause a maladaptive reorganization of RS functional brain networks. During even more chronic phases of complete SCI, this continued disruption of efferent and afferent pathways was portrayed in an overall decrease in FC (Oni-Orisan et al., 2016). The sensorimotor networks of SCI patients and healthy individuals seem to share similar patterns of connectivity but a few new functional interactions have been identified as unique to SCI patient networks (Mattia et al., 2009). These interactions that we previously described could be attributed to both adaptive and maladaptive organization effects after the injury (Mattia et al., 2009; De Vico Fallani et al., 2010), the importance of which as possible prognostic factors or their effect on patient rehabilitation cannot be yet clarified. On the other hand, Chisholm et al. (2015) have also provided some indication about the importance of residual reciprocal communication of sensorimotor pathways during chronic phases. The authors published a case report of a 31-year-old individual with incomplete SCI 10 years before their intervention. Using RS fMRI correlation analysis they evaluated an increase in local RS FC of the right M1 (leg region) and decreased connection with left M1 after rehabilitation sessions, while spatial pattern of connectivity remained unchanged. With regards to his neurological condition the patient had higher residual function of the left lower extremity than of the right. While only a single case report, such findings denote that adaptive network plasticity could possibly be summoned through rehabilitation even in long-term injury (a decade after the initial injury) but it is also important to consider individual differences in rehabilitation outcomes (Whiteneck et al., 2009). Moreover, single-subject fMRI connectivity analysis is generally considered too underpowered and non-linear dynamic methods are proposed to provide more sound conclusions (Rădulescu and Hannon, 2017).

Intrinsic spinal cord plasticity in non-injured individuals has also been demonstrated (Vahdat et al., 2015) but the exact underlying neurophysiological process and the extent that this is modulated by higher-order interactions is also not fully understood. There are several indications that higher-order interactions are driven by SCI as a compensatory mechanism. Hou et al. (2016) identified a pattern: patients that 6 months later presented good recovery had already developed an adaptive increase of interhemispheric communication at the sub-acute phase, while those patients that later presented bad recovery had developed a maladaptive change of decreased interhemispheric communication (Hou et al., 2016). Nonetheless, it is yet unclear whether those early changes represent an active attempt of the CNS to overcome the impairment of the long neural pathways or they are merely reflective of the underlying injury. Most studies that were included in this qualitative synthesis investigated FC on chronic SCI patients with complete injury and thus were unable to provide insight regarding the dynamics of the process. We can identify important limitations of the existing literature and of the methods used at large: due to the nature and gravity of the condition, it is very difficult to design a study to include patients at the acute phase of injury and virtually impossible to have pre-injury recordings of the baseline of those patients. Also, due to the small number of published studies and the variety of connectivity metrics used among them, no further quantitative study (meta-analysis) was possible, that could otherwise help in producing more informative results.

## Conclusion

Although important insight is provided, existing literature cannot yet precisely explain the pathophysiological process and effect of SCI on the connectivity of the brain. Studies needed to address this issue could be on a larger scale and of a longitudinal design, also combining more than one neurophysiological recording method in patients after SCI. This should be considered in order to better understand how reorganization of the brain network is installed and progresses through the course of the condition. We can identify specific questions that need to be answered through further investigation using such a proposed design: (a) how and why reorganization of brain networks is established; (b) how this reorganization evolves in time with respect to the severity and chronicity of the injury; (c) when can it be considered an adaptive or maladaptive evolution; and (d) how can it be promoted or prevented respectively. Simultaneous analysis of activations of the brain and spinal cord as well as their interactions (connectivity) could also shed further light on this subject. The expected insight could hold clinical relevance in preventing maladaptive plasticity after SCI through individualized neuro-rehabilitation, as well as in the design of connectivity-based brain-computer interfaces and assistive technologies for SCI patients (Athanasiou et al., 2016b, 2017; Hamedi et al., 2016; Pazzaglia and Molinari, 2016).

## Definitions

- Brain/Cortical connectivity: a pattern of interactions between different brain (or only cortical) regions that can be based on neuroanatomical connections, functional or causal statistical dependencies (Astolfi et al., 2007)
- Characteristic path length: a metric of the shortest possible distance between pairs of nodes of a network according to graph theory, measuring the network’s integration (Watts and Strogatz, 1998)
- Clustering coefficient: a metric of the network’s segregation into different clusters of nodes or modules according to graph theory (Watts and Strogatz, 1998)
- Directed Transfer Function: a connectivity metric; multivariate spectral EEG technique used to estimate directional causality between activation time series of network nodes (Sinatra et al., 2009)
- Functional (brain/cortical) connectivity: a pattern of dynamic interactions between different specialized brain regions, not limited to anatomical connections, measured by functional neuroimaging techniques such as EEG or fMRI (Sinatra et al., 2009)
- Graph analysis: a method based on theoretical mathematical framework (graph theory) used to compute, analyze and represent interactions between nodes of a network (Watts and Strogatz, 1998)
- Intrinsic spinal cord connectivity: a pattern of functional neurophysiological interactions organized intrinsically within the level of the spinal cord (Vahdat et al., 2015)
- Local/global efficiency: a measure of how efficiently the network shares information at local cluster level or in total; an alternative to the clustering coefficient/characteristic path length scheme (Latora and Marchiori, 2001)
- Markov Clustering: an algorithm that simulates random paths over a network, used to detect structures of densely connected nodes on directed information flow patterns (Sinatra et al., 2009)
- Modularity: a metric of the network’s segregation into modules; see clustering coefficient
- Network-based statistics: a statistical method used to identify significant interactions within a network, able to reduce mass-univariate testing-related statistical errors (Zalesky et al., 2010)
- Partial Directed Coherence: a connectivity metric; multivariate spectral EEG technique used to estimate directional causality between activation time series of network nodes (Astolfi et al., 2007)
- Redundancy index: a metric of the network’s robustness. Three different redundancy indexes have been defined: (a) scalar redundancy which refers to the global redundancy of the network; (b) vector redundancy that characterizes the total redundancy for each path length; and (c) matrix redundancy which is the redundancy in each pairs of nodes independently of the path length.
- Small-world-ness: a network topology characterized by high clustering but low characteristic path length, a model with increased signal-propagation speed and computational power, commonly featured in biological systems, among others (Watts and Strogatz, 1998).

## Author Contributions

AA designed the study and wrote the article. AA, MAK and NP performed the search, removed the duplicates and screened the records. AA, MAK, NP, NF and KRK read and assessed the full-texts for eligibility. KP and PDB contributed to the interpretation of the results. All authors read and revised the manuscript drafts. All authors read and approved the final manuscript version. PDB is the guarantor of the study.

## Conflict of Interest Statement

The authors declare that the research was conducted in the absence of any commercial or financial relationships that could be construed as a potential conflict of interest.

## Abbreviations

ALFF: Amplitude of Low Frequency Fluctuations
BG: Basal Ganglia
CB: Cerebellum
CI: Confidence Interval
CMA: Cingulate Motor Area
EEG: Electroencephalography
FC: Functional Connectivity
fMRI: functional Magnetic Resonance Imaging
M1: Primary Motor Area
OFC: Orbito-Frontal Cortex
PMC: Pre-Motor Cortex
ROI: Region of Interest
RS: Resting State
S1: Primary Somatosensory Area
S2: Secondary Somatosensory Area
SCI: Spinal Cord Injury
SMA: Supplementary Motor Area
SPC: Superior Parietal Cortex
TH: Thalamus.
